# Diagnostic utility of a line probe assay for multidrug resistant-TB in smear-negative pulmonary tuberculosis

**DOI:** 10.1371/journal.pone.0182988

**Published:** 2017-08-22

**Authors:** Binit Kumar Singh, Surendra K. Sharma, Rohini Sharma, Vishnubhatla Sreenivas, Vithal P. Myneedu, Mikashmi Kohli, Dinkar Bhasin, Sanjay Sarin

**Affiliations:** 1 Department of Internal Medicine, All India Institute of Medical Sciences, New Delhi, India; 2 Department of Biostatistics, All India Institute of Medical Sciences, New Delhi, India; 3 National Institute Tuberculosis and Respiratory Diseases, New Delhi, India; 4 Foundation for Innovative New Diagnostics, New Delhi, India; Indian Institute of Technology Delhi, INDIA

## Abstract

**Objective:**

To evaluate the performance of Genotype MTBDRplus VER 2.0 in the diagnosis of *Mycobacterium tuberculosis* (MTB) in sputum smear-negative pulmonary TB cases.

**Methods:**

A total of 572 Ziehl-Neelsen sputum smear-negative samples were selected and subjected to line probe assay (Genotype MTBDRplus VER 2.0), and culture in mycobacterial growth indicator tube (MGIT-960). Immunochromatographic test was used to confirm the MTB-complex (MTBC) in culture-positive samples and phenotypic drug-susceptibility testing was done using MGIT-960.

**Results:**

The line probe assay was able to diagnose MTBC in 38.2% (213/558) of specimens after excluding 14 nontuberculous mycobacteria. Sensitivity and specificity of the assay were 68.4% and 89.3% respectively, considering MGIT-960 culture as gold standard after excluding contaminated and invalid results. On comparing with composite reference standard, the assay had 71.5% sensitivity and 100% specificity in the diagnosis of tuberculosis. The sensitivity and specificity for detecting resistance to rifampicin (RMP) were 100% and 99.24% respectively and for resistance to isoniazid (INH) were 97.62% and 98.55%, respectively.

**Conclusion:**

Genotype MTBDRplus VER 2.0 is a rapid and precise diagnostic tool for detection of MTB in sputum smear-negative samples. It also facilitates accurate diagnosis of RMP and INH resistance within turn around-time.

## Introduction

Pulmonary tuberculosis (PTB) is a major public health disease that ranks as the second-most leading cause of death worldwide, after human immunodeficiency virus infection. In 2016, there were 10.4 million new PTB cases globally, with 1.4 million deaths. From a public health prospective, early and rapid diagnosis of PTB is important because of its potential for transmission [1].

Previously, solid media based conventional drug susceptibility testing (DST) was performed for detection of drug resistant *Mycobacterium tuberculosis* (MTB), which could take months to confirm results. After the introduction of broth-based media DST, the results time reduced to weeks. However, the contamination rate limits its utility in a resource-limited setting. To overcome these limitations, in 2008, the World Health Organization (WHO) certified and endorsed the line probe assay (LPA) as a molecular method for the rapid diagnosis of TB and simultaneous detection of rifampicin (RMP) and isoniazid (INH) resistance [2]. However, the previous version of LPA (GenoType MTBDRplus version 1.0) has been evaluated only for high-grade smear-positive specimens and to detect the level of drug resistance in culture isolates. Therefore, the assay can be used for smear-negative sputum samples and scanty positive samples (i.e. those with bacillary loads less than 10 bacilli in 100 fields of the smear) only after isolation becomes culture-positive [3,4]. The latest version of LPA (GenoType MTBDRplus version 2.0, Hain Lifescience, Nehren, Germany) was introduced to overcome these limitations. This new version can detect MTB and their DST patterns for the two first-line drugs RMP and INH in smear-positive (including scanty), smear-negative, and culture-positive samples [5].

The present study aimed to evaluate the diagnostic performance of LPA version 2.0 to detect MTB in smear-negative PTB cases. For smear-negative PTB samples, the sensitivity and specificity of LPA is suboptimal as compared to Mycobacterial Growth Indicator Tube (MGIT)-960 cultures [6]; therefore, a composite reference standard (CRS) was used to evaluate LPA in this study.

## Materials and methods

### Ethics approval

The study was approved by the institutional ethics committee of the All India Institute of Medical Sciences (AIIMS), New Delhi, India.

### Subject recruitment

Patients were recruited from the Directly Observed Treatment Short-Course (DOTS) centres of six chest clinics in four districts of Delhi and the DOTS centre and Medical Out-Patient Department of the AIIMS hospital. Written informed consent was obtained from all patients, or their legal representatives, at the time of enrolment into the study (October 2012 to May 2013). All investigations were conducted at the Intermediate Reference Laboratory (IRL) of the Department of Medicine at AIIMS, New Delhi, India. Since 2011, the IRL has been accredited for LPA (molecular DST) and MGIT-960 (DST) by the National Mycobacteriology Accreditation System of the Central TB Division of the Ministry of Health and Family Welfare, Government of India.

Sputum samples from all patients suspected for TB and those receiving retreatment were subjected to smear microscopy by Ziehl–Neelsen (ZN) staining and liquid culture (LC) as part of the routine diagnostic protocol. Only smear-negative samples were included in the study and further tested by LPA. An immunochromatographic test (ICT) was performed for all culture-positive samples to rule out non-tuberculous mycobacteria (NTM), using an SD BIOLINE TB Ag MPT64 kit (SD Bioline, Standard Diagnostics, Suwon, Korea) [7]. To minimize bias, all laboratory technicians performing the molecular and reference tests were aware only of their respective laboratory results.

### Specimen processing

Two sputum samples (spot and next day morning) from all patients suspected for TB were collected in 50-mL wide-mouthed sterile falcon tubes according to the guidelines of the Revised National Tuberculosis Control Programme of India [8]. The sputum specimens were processed in a type II bio-safety cabinet at a bio-safety level-3 laboratory. Before decontamination, sputum samples were subjected to direct smear microscopy, using the ZN method. The samples were decontaminated by the NALC-NaOH method (final NaOH concentration, 1%) [9]. After decontamination, the spot samples were inoculated in LC and the morning samples were subjected to LPA.

### Liquid culture

An MGIT-960 tube was prepared by adding 0.8 mL oleic acid–albumin-dextrose-catalase PANTA (Becton Dickinson). A 0.5-mL volume of processed sample was inoculated into the respective tube and incubated at 37°C in an automated BACTEC MGIT-960 (Becton Dickinson) instrument for a maximum period of 42 days, with scheduled regular monitoring. The time-to-positivity was recorded for the positive-flagged MGIT-960 tubes. ZN microscopy was performed to visualise the serpentine cord formation (typical tight, rope-like aggregates morphology that MTB exhibit during growth in liquid media) along with sterility on brain heart infusion agar media [10]. Indirect DST was performed using the final critical concentration of anti-TB drugs, i.e. 0.1 μg/ml INH and 1.0 μg/ml RMP [11].

### LPA

Mycobacterial DNA was extracted using a GenoLyse kit (Hain Lifescience, Nehren, Germany)-based manual method; however, instead of the 0.5 mL recommended by the manufacturer, we used 1 mL of decontaminated sputum sample. Polymerase chain reaction (PCR) was performed using pre-made amplification mixes (amplification mix A and amplification mix B) that contained all the necessary components. Hybridization was performed using an automated GT Blot 48 device (Hain Lifescience, Nehren, Germany), and the results were interpreted based on the operating manual provided by the manufacturer [5, 6].

### Patient categorization

On the basis of a CRS algorithm (S1 Table), the patients were categorized into two groups: TB cases [either culture-positive or culture-negative with other evidence, such as clinical symptoms and radiological findings, and response to anti-tuberculosis treatment (ATT) after 2 months of being positive] and non-TB cases (clinically suspected of TB; culture may be positive or negative but other evidence was negative and the patients did not respond to ATT.

### Statistical analysis

Statistical analysis was performed using Stata 11.2 (College Station, TX, USA). All data are presented as frequency (percentage) and mean (± SD). CRS was used as the gold standard to evaluate the sensitivity, specificity, positive predictive value (PPV), and negative predictive value (NPV) based on the culture results and the clinical and radiological findings.

## Results

**Fig 1:**
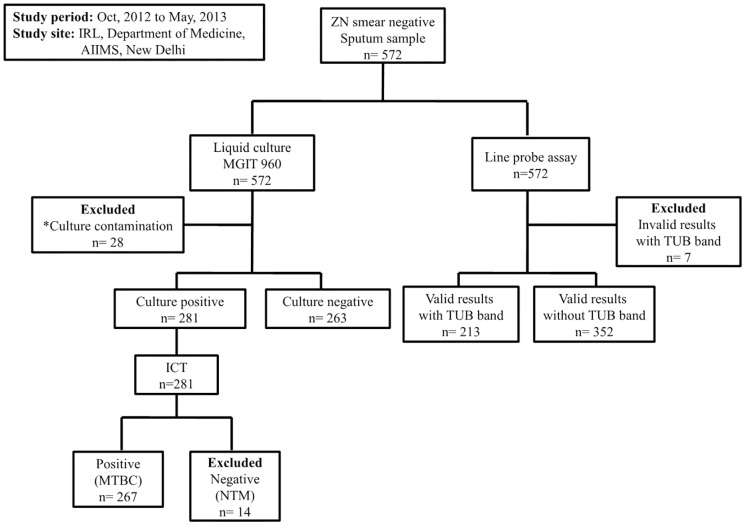
Schematic representation of work flow. ZN: Ziehl–Neelsen, MGIT: Mycobacteria Growth Indicator Tube, MTBC: *Mycobacterium tuberculosis* complex, NTM: non-tuberculous mycobacteria, ICT: Immunochromatographic test, Valid result with TUB band, LPA strips are interpretable with MTB complex band; Invalid result with TUB band, LPA strips are not interpretable with MTB complex band; Valid result without TUB band, LPA strips without MTB complex band; * one contaminated culture was found to invalid results with TUB.

A total of 572 sputum smear-negative PTB suspects, including retreated patients, were recruited in the study. The mean age of the patients was 34.4 years (SD, 15.5 years); 341 of the patients were male and 231 were female. Forty-eight specimens from the patients were excluded from the study because they produced invalid results with TUB band (it represents the presence of the *M*. *tuberculosis* complex in LPA strips), culture contamination, and the presence of NTMs (Fig 1 and S1 Fig)

Among all the patients’ specimens, 281 (49.1%) were found to be culture-positive, 263 (46%) were culture-negative, and 28 (4.9%) showed contamination as assessed by LC method. For the LPA in culture-positive samples, 180 (32.3%) showed valid results with TUB bands (S1 Fig); 83 (14.9%) yield valid results without TUB bands. Among the culture-negative and contaminated samples, 28 (5%) and five (0.9%) showed valid results with TUB bands, respectively (S2 Table). The sensitivity and specificity of this LPA kit to detect MTB (TUB bands) in all smear-negative samples with culture as a reference standard were 68.4%; 180/263 (95% CI: 62.5–74%) and 89.3%; 233/261 (95% CI: 84.9–92.8%), respectively (Table 1). However, the LPA detected MTB in 213 (38.2%) samples, of which 180 (32.3%) were culture-positive, 28 (5%) were culture-negative, and five (0.9%) were contaminated (S2 Table). Therefore, when the LPA was compared with the CRS algorithm, its sensitivity and specificity increased to 71.5% (208/291; 95% CI: 65.9–76.6%) and 100% (233/233; 95% CI: 93.2–100%), respectively.

**Table 1:** Diagnostic accuracy of LPA version 2.0 with and without CRS.

Method	Performance (%), No. of positive samples/ total no. of samples (95% CI)			
	Sensitivity	Specificity	PPV	NPV
**Genotype MTBDR plus VER 2.0 (culture as reference standard)**	68.4, 180/263 (62.5–74)	89.3, 233/261 (84.9–92.8)	86.5, 180/208 (81.1–90.9)	73.7, 261/316 (68.5–78.5)
**Genotype MTBDR plus VER 2.0 (CRS as reference standard)**	71.5, 208/291 (65.9–76.6)	100, 233/233 (93.2–100)	100, 208/208 (98.2–100)	73.7, 233/316 (68.5–78.5)

NPV: negative predictive value; PPV: positive predictive value; CRS: Composite Reference Standard

The performance of the LPA to detect RMP and INH resistance was evaluated by comparing it with the LC (MGIT-960)-based DST results (Table 2). The sensitivity and specificity of LPA to detect RMP resistance were 100% and 99.2%, whereas those to detect INH resistance were 97.6% and 98.6%, respectively.

**Table 2:** Performance parameter of LPA version 2.0.

MTBDR plus VER 2.0	DST result (MGIT 960) (n: 180)				Performance % (95% CI)			
	RMPr	RMPs	INHr	INHs	Sensitivity	Specificity	PPV	NPV
**RMPr**	49	01	-	-	100 (92.6–100)	99.2 (95.8–99.9)	98 (89.3–99.7)	100 (97.2–100)
**RMPs**	00	130	-	-				
**INHr**	-	-	41	02	97.6 (87.3–99.6)	98.6 (94.8–99.7)	95.4 (84.5–99.7)	99.3 (95.2–99.4)
**INHs**	-	-	01	136				

RMPr: resistant to rifampin, RMPs: susceptible to rifampin, INHr: resistant to isoniazid, INHs: susceptible to Isoniazid, NPV: negative predictive value, PPV: positive predictive value, Only 180 samples have both MGIT DST and genotypic DST results and hence were included.

The frequency of mutations was calculated based on all valid LPA results with TUB bands (Table 3, n = 213). In total, 59 (27.6%) strains were RMP-resistant and the most widespread mutation in *rpoB* was the S531L amino acid substitution (n = 43, 72.9%), followed by mutations in codons 530–533 (n = 6, 10.1%), codons 526–529 (n = 3, 5.1%), codons 513–519 (n = 3, 5.1%), D516V (n = 1, 1.7%), codons 510–517 (n = 1, 1.7%), codons 510–513 (n = 1, 1.7%), and codons 505–509 (n = 1, 1.7%). Fifty-two (24%) strains were INH-resistant. Among them, 44 were *katG* resistant, four were *inhA* resistant, and four were both *katG* and *inhA* resistant. The most prevalent mutation in *katG* resulted in an amino acid substitution (S315T) in 45 (86.5%) strains. Three (5.8%) strains had unknown amino acid substitution mutations, three (5.8%) had an *inhA* C15T amino acid substitution, and one (1.9%) had an unknown amino acid substitution mutation at the -15 position of *inhA* (Table 3).

**Table 3:** Pattern of gene mutations in *Mycobacterium tuberculosis* strains using LPA version 2.0.

**Mutation pattern of RIF (*rpoB*) obtained from Genotype MTBDR plus V.2 assay (59/213)**				
**Gene**	**Band**	**Mutation point**	**No. of samples**	**Frequency (%)**
*rpoB* (n:59)	WT1	505–509	1	1.7
	WT2	510–513	1	1.7
	WT2/ WT3	510–517	1	1.7
	WT3/ WT4	513–519	3	5.1
	WT7	526–529	3	5.1
	WT8	530–533	6	10.1
	MUT1	D516V	1	1.7
	MUT3	S531L	43	72.9
**Mutation pattern of INH (*katG* & *inhA*) obtained from Genotype MTBDR plus V.2 assay (52/213)**				
*katG* (n:48)	WT	315	3	5.8
	MUT1	S31T	45	86.5
*inhA* (n:4)	WT	-15	1	1.9
	MUT1	C15T	3	5.8

WT: wild type probe, MUT: mutation probe, C: cysteine, D: aspartic acid, L: leucine, S: serine, T: threonine, V: valine, all 213 results of LPA were included.

## Discussion

For several decades, ZN smear microscopy has been the mainstay in the diagnosis of PTB, particularly in resource-limited, high-TB-burden countries such as India [12]. However, the technique has low sensitivity [13], is highly observer-dependent, and is incapable of distinguishing between MTBC and NTM strains. In 2009, following the WHO recommendation, fluorescent light-emitting diode (LED) microscopy, which has a higher sensitivity to detect TB bacilli, was intended to replace ZN microscopy. However, until 2014, only 7% of microscopy centres globally were equipped with LED microscopes [14]. Hence, several rapid diagnostic tests have emerged for PTB diagnosis. Here, we evaluated the utility of the latest LPA (version 2.0) for the rapid diagnosis of sputum smear-negative PTB.

The aim of our study was to evaluate the utility of the latest version of LPA for the diagnosis of sputum smear-negative PTB patients. Almost similar sensitivity (66.7%) with high specificity (92.3%) was observed in a study conducted by Ninan et al. [15] for the detection of MTB with MGIT-960 culture as reference standard. Another study conducted by Meaza et al. [16] showed both higher sensitivity (77.8%) and specificity (97.9%). The sensitivity from these studies is comparable to the present study, however, higher specificity of these previous studies could be attributed to the fact that they had very small sample size for smear negative sample (n = 29 and n = 18 respectively).

CRS is a new concept used to evaluate the diagnostic accuracy of any test, especially in TB. It takes into account the clinical symptoms, other available tests, and response to treatment to define correctly whether a case is TB. In the comparison with CRS, we observed an increase in the sensitivity and specificity of the LPA, compared to that in comparison with MGIT-960. Twenty-eight cases were culture negative but TB positive according to the CRS algorithm. LPA produced valid results for these 28 cases, of which nine were multidrug resistant tuberculosis (MDR-TB) cases. This is important; because the undiagnosed patients would otherwise not have received treatment based on acid-fast Bacilli and culture-negative reports, and would have further led to increased morbidity and mortality.

Previous studies conducted using LPA version 1.0 reported much lower detection rates of MTBC (only 14–16%) in smear-negative specimens [17]. This discrepancy could be because LPA version 2.0 uses a Genolyse kit for MTB genomic DNA isolation, instead of the mechanical methods used by version 1.0, which decreases the chance of cross-contamination and increases the quality of the amplified genomic DNA, producing better results. Moreover, in our study, we used larger volumes of decontaminated sputum sample (1 mL) as compared to that recommended by the manufacturer (0.5 mL). This might have resulted in higher detection rates of MTB by LPA from the previous studies conducted by Ninan et al [15] and Meaza et al [16]; however, it warrants validation in future studies and the possible re-evaluation of the assay’s methodology.

LPA not only detects the presence of MTBC in the samples, but also reveals the resistance pattern against two first-line drugs, RMP and INH, with high accuracy. The sensitivity and specificity of the newer LPA to detect RMP and INH susceptibility was the same as that reported previously (Table 2) [18,19]. For RMP resistance, excellent percentage accuracy was found in the detection of mutation related to 81 bp region of *rpoB* gene [17] but for INH resistance, low accuracy was observed [17, 20]. The possible reason for very few discrepant and low accuracy results could have been caused by some masked mutations in the genomic regions of *rpoB*, *katG*, and *inhA* [21,22].

To differentiate between MTBC and NTM using ICT on culture samples, in the present study LPA revealed a specificity of 100% and these findings are in agreement with those of previously published studies [9, 23, 24]. According to an Indian study, the range of NTM varies from 0.7 to 34% in PTB samples [25]. Our study reflected the percentage of NTM (2.5%) within this range.

In our study, LC (MGIT-960) was used as a reference standard along with CRS to detect MTB, because of its higher culture-positivity rates compared with conventional solid culture medium (LJ medium). Furthermore, LC has the added advantage of shorter turn-around time [26]. The proportion of invalid results was significantly lower, i.e., 1.3% with LPA version 2.0, as compared to contamination rates of LC (5%).

The landscape of TB diagnosis has changed over the past few years because of the emergence of rapid diagnostic tests, such as LPA and Xpert MTB/RIF. LPA has a short turn-around time of 24–48 hours to detect MTB and the simultaneous detection of RIF and INH resistance. Moreover, Xpert MTB/RIF only reports the RMP sensitivity status [27]. In the present study, 13 samples were RMP-sensitive and INH-resistant. Such patients would be missed if only Xpert MTB/RIF is used. Furthermore, 20 of the culture isolates were RMP-resistant and INH-sensitive. Such patients are likely to be falsely classified as having MDR-TB, if only Xpert MTB/RIF is used, and could lead to overestimation of MDR-TB cases. Nevertheless, there are a few major drawbacks of LPA. These include its high operational costs, requirement for skilled technicians, and the need for regular maintenance.

These findings suggest that even though the WHO does not recommend LPA for smear-negative PTB samples, optimal diagnostic accuracy was observed in the present study. Hence, LPA may be a useful diagnostic tool to detect smear-negative PTB cases with additional drug susceptibility testing for both RIF and INH.

## Conclusion

In conclusion, the latest version of LPA has an optimal sensitivity of 71.5% to detect MTBC in smear-negative sputum samples. It also provides rapid information on drug susceptibility of MTBC to RMP and INH. This test can be recommended as a rapid molecular diagnostic test for sputum smear-negative pulmonary TB cases. Future studies should focus on confirming these findings in a large patient cohort.

## Supporting information

S1 Table Diagnostic algorithm under composite reference standard (CRS) for patients’ categorization. (DOCX)

S2 Table Diagnostic accuracy of Genotype MTBDR plus VER 2.0 with liquid culture (BACTEC MGIT-960). (DOCX)

S1 Fig Schematic representation of the study design. (TIFF)
