# Supplementary material for: Diagnostic utility of a line probe assay for multidrug resistant-TB in smear-negative pulmonary tuberculosis
Source: PLoS One. 2017 Aug 22;12(8):e0182988. doi: 10.1371/journal.pone.0182988 (PMC5568731; doi:10.1371/journal.pone.0182988)
Supplement: S1 Table — (DOCX) [file pone.0182988.s001.docx]

**S1 Table: Diagnostic algorithm under composite reference stander (CRS) for patients’ categorization**

| **Condition** | **Liquid Culture** | **Symptoms** | **Radiology** | **Follow-up** |
| --- | --- | --- | --- | --- |
| **TB**  **cases** | + | + | +/- | + |
|  | - | + | + | + |
|  | - | + | - | + |
| **Non-TB**  **cases** | - | + | - | - |
|  | + (in case of NTM) | + | - | - |

NTM :non-tuberculous mycobacteria, + :Disease positive; - :Disease negative; +/- : Disease positive/negative
