# Supplementary material for: Diagnostic utility of a line probe assay for multidrug resistant-TB in smear-negative pulmonary tuberculosis
Source: PLoS One. 2017 Aug 22;12(8):e0182988. doi: 10.1371/journal.pone.0182988 (PMC5568731; doi:10.1371/journal.pone.0182988)
Supplement: S2 Table — (DOCX) [file pone.0182988.s002.docx]

**S2 Table: Diagnostic accuracy of Genotype MTBDR plus VER 2.0 with liquid culture (BACTEC MGIT-960)**

| **MTBRplus VER 2.0** | **BACTEC MGIT 960** | | | | **Performance % (95% CI)** | | | |
| --- | --- | --- | --- | --- | --- | --- | --- | --- |
|  | **Culture +ve** | **Culture -ve** | **Contamination** | **Total** | **Sensitivity** | **Specificity** | **PPV** | **NPV** |
| Valid result with TUB band | 180 (32.3%) | 28 (5%) | 5 (0.9%) | 213 (38.2%) | 68.4, 180/263 (62.4- 74) | 89.3, 233/261 (84.9-92.8) | 86.5, 180/208 (81.1-90.9) | 73.7 261/316 (68.5-78.5) |
| Invalid result with TUB band | 4 (0.7%) | 2 (0.4%) | 1 (0.2%) | 7 (1.3%) |  |  |  |  |
| Valid result without TUB band | 83 (14.9%) | 233 (41.6%) | 22 (3.9%) | 338 (60.6%) |  |  |  |  |
| Total | 267 (47.8%) | 263 (47.1%) | 28 (5.0%) | 558 |  |  |  |  |

PPV: Positive Predictive Value, NPV: Negative Predictive Value, 14 NTM were excluded from this table; Valid result with TUB band, LPA strips are interpretable with MTB complex band; Invalid result with TUB band, LPA strips are not interpretable with MTB complex band ; Valid result without TUB band, LPA strips without MTB complex band
