# Supplementary figures and images for: Diagnostic utility of a line probe assay for multidrug resistant-TB in smear-negative pulmonary tuberculosis
Source: PLoS One. 2017 Aug 22;12(8):e0182988. doi: 10.1371/journal.pone.0182988 (PMC5568731; doi:10.1371/journal.pone.0182988)

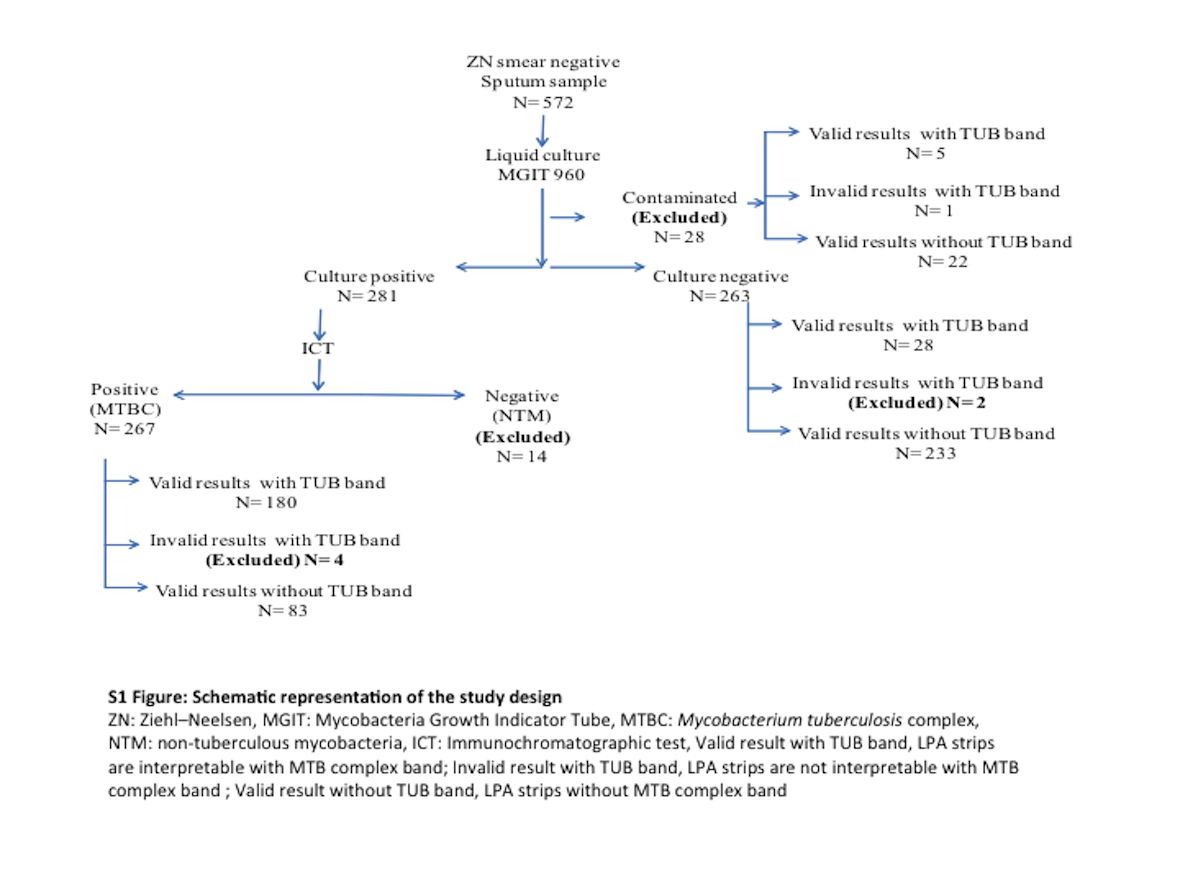

Supplement: S1 Fig — (TIFF) [file pone.0182988.s003.tiff]
